# High-density genetic map construction and gene mapping of pericarp color in wax gourd using specific-locus amplified fragment (SLAF) sequencing

**DOI:** 10.1186/s12864-015-2220-y

**Published:** 2015-12-09

**Authors:** Biao Jiang, Wenrui Liu, Dasen Xie, Qingwu Peng, Xiaoming He, Yu’e Lin, Zhaojun Liang

**Affiliations:** Vegetable Research Institute, Guangdong Academy of Agriculture Science, Guangzhou, 510640 China; Guangdong Provincial Key Lab for New Technology Research on Vegetables, Guangzhou, 510640 China

**Keywords:** SLAF-seq, Wax gourd, Genetic map, Mapping, Pericarp color

## Abstract

**Background:**

High-density map is a valuable tool for genetic and genomic analysis. Although wax gourd is a widely distributed vegetable of *Cucurbitaceae* and has important medicinal and health value, no genetic map has been constructed because of the lack of efficient markers. Specific-locus amplified fragment sequencing (SLAF-seq) is a newly developed high-throughput strategy for large-scale single nucleotide polymorphism (SNP) discovery and genotyping.

**Results:**

In our present study, we constructed a high-density genetic map by using SLAF-seq and identified a locus controlling pericarp color in wax gourd. An F2 population of 140 individuals and their two parents were subjected to SLAF-seq. A total of 143.38 M pair-end reads were generated. The average sequencing depth was 26.51 in the maternal line (B214), 27.01 in the parental line (B227), and 5.11 in each F2 individual. When filtering low-depth SLAF tags, a total of 142,653 high-quality SLAFs were detected, and 22,151 of them were polymorphic, with a polymorphism rate of 15.42 %. And finally, 4,607 of the polymorphic markers were selected for genetic map construction, and 12 linkage groups (LGs) were generated. The map spanned 2,172.86 cM with an average distance between adjacent markers for 0.49 cM. The inheritance of pericarp color was also studied, which showed that the pericarp color was controlled by one single gene. And based on the newly constructed high-density map, a single locus locating on chromosome 5 was identified for controlling the pericarp color of wax gourd.

**Conclusions:**

This is the first report of high-density genetic map construction and gene mapping in wax gourd, which will be served as an invaluable tool for gene mapping, marker assisted breeding, map-based gene cloning, comparative mapping and draft genome assembling of wax gourd.

**Electronic supplementary material:**

The online version of this article (doi:10.1186/s12864-015-2220-y) contains supplementary material, which is available to authorized users.

## Background

Wax gourd (*Benincasa hispida* (Thunb.) Cogn, 2n = 2*x* = 24) is a monotypic genus, which belongs to *Cucurbitaceae* and is a widely distributed vegetable in India, China and many other tropical countries [1]. It is also called white gourd, white pumpkin, tallow gourd, and so on, which is named after an Italian count, Giuseppe Benincasa [2]. Fruit is its main consumption organ, and often consumed as baked, fried, boiled, pickled and candied/preserved. The storage of wax gourd is quite long, which makes it play a significant role in ensuring the annual supply and regulating off-seasons of vegetables. Besides, it is also recommended for treatment of peptic ulcer, hemorrhages from internal organs, epilepsy and other neurological disorders [3, 4]. Previous study also showed that its fresh juice is effective in preventing morphine withdrawal in mice [5]. In spite of its economic importance, no genetic map has been constructed in wax gourd, which hinders the map-based cloning of important horticultural genes.

High-density genetic maps are particularly valuable tools in many genetic and genomic applications, especially for gene fine mapping and map-based gene cloning. One key prerequisite for high-density genetic map construction is the available of a large number of polymorphic markers. In wax gourd, only conventional markers such as random amplified polymorphic DNA (RAPD) and simple sequence repeats (SSR) could be available and had been used in genetic analysis [6, 7]. And recently, we have carried out transcriptome sequencing and developed thousands of SSR markers in wax [8]. Because of the narrow genetic base, no genetic map has been constructed in wax gourd. Besides, the genome size of wax gourd reaches up to 800 Mb (unpublished observations), which is much bigger than any of the known species in cucurbits, such as cucumber (367 Mb), melon (450 Mb), watermelon (425 Mb) [9–12]. Consequently, the existing markers make it impossible for constructing high-density genetic map. SNP (single nucleotide polymorphism) is more useful than other conventional markers, because it is the most abundant and stabile type of genetic variation in most genomes [13–15]. However, the difficulty is the development of a large number of SNP markers, which limits the application of this kind of markers.

Fortunately, with the development of biotechnology, the next-generation sequencing (NGS) technology makes it feasible for large scale SNP marker discovery. Reduced representation genome sequencing (RRGS) is a rapid and cost-effective strategy for high-throughput identification and genotyping of SNPs [16, 17]. This sequencing method reduces the genome complexity by only sequencing the DNA fragments with restriction sites. Accompanying with the extensive application of NGS technology, several RRGS methods have been developed [18, 19]. SLAF-seq is a recently developed method which is a low cost but efficient high-throughput sequence-based technique and reduces the complexity of high-quality reference genome libraries [20]. This technology exhibits significant advantages in high-throughput SNP marker discovery and genotyping for constructing genetic map. It has been employed for high-density genetic map construction in many species, such as common carp [20], sesame [21], soybean [22, 23], mei [24] and cucumber [25, 26].

Since SLAF-seq technique does not depend on reference genome sequence, it is particularly important for species with unknown genome, including wax gourd. In the current study, the SLAF-seq technique was employed to construct an SNP-based genetic map for wax gourd, which contained 12 linkage groups and 4,607 SNP markers, and spanned 2,172.86 cM with an average marker interval of 0.47 cM. In addition, to text the utility of this genetic map, fine mapping of pericarp color trait was detected. This is the first report of genetic map construction in wax gourd.

## Results

### SLAF sequencing and genotyping

**Fig. 1:**
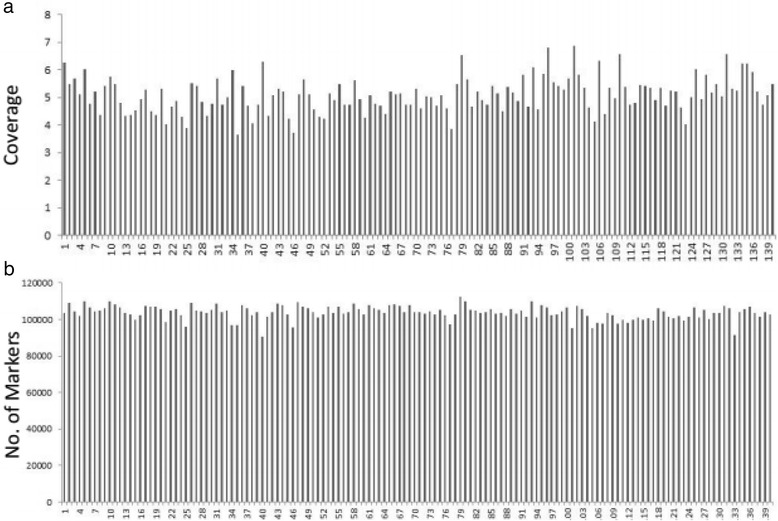
Number of markers and average sequencing depths of F2 population. The x-axes in both a and b indicate the F2 individuals. The y-axe in a indicates marker coverage, and the y-axe in b indicates the number of markers developed for each F2 plant

After SLAF library construction and high-throughput sequencing, 143.38 M pair-end reads was obtained with a length of 100 bp. The guanine-cytosine (GC) content as 37.46 % and Q30 ratio (a quality score of 30) was 83.47 %. In the maternal line (B214), 6,137,771 reads and 128,948 SLAFs with an average coverage of 26.51 were generated. In the parental line (B227), the number of reads and SLAFs were 6,069,781 and 129,256, respectively, with an average depth of 27.01 for each SLAF. For F2 population, 90,367 to 112,548 SLAFs were produced from 643,155 to 1,297,034 reads, with the coverage ranged from 3.66 to 6.89. And the average number of reads per individual was 1,009,705, with an average depth of 5.11 (Fig. 1). The average number of SLAFs was 103,716 (Table 1).

**Table 1:** Summary of SLAF depths

Samples	SLAF numbers	Total depth	Average depth
B214	128,948	3,418,551	26.51
B227	129,256	3,491,290	27.01
Offspring	103,716	529,675	5.11

**Fig. 2:**
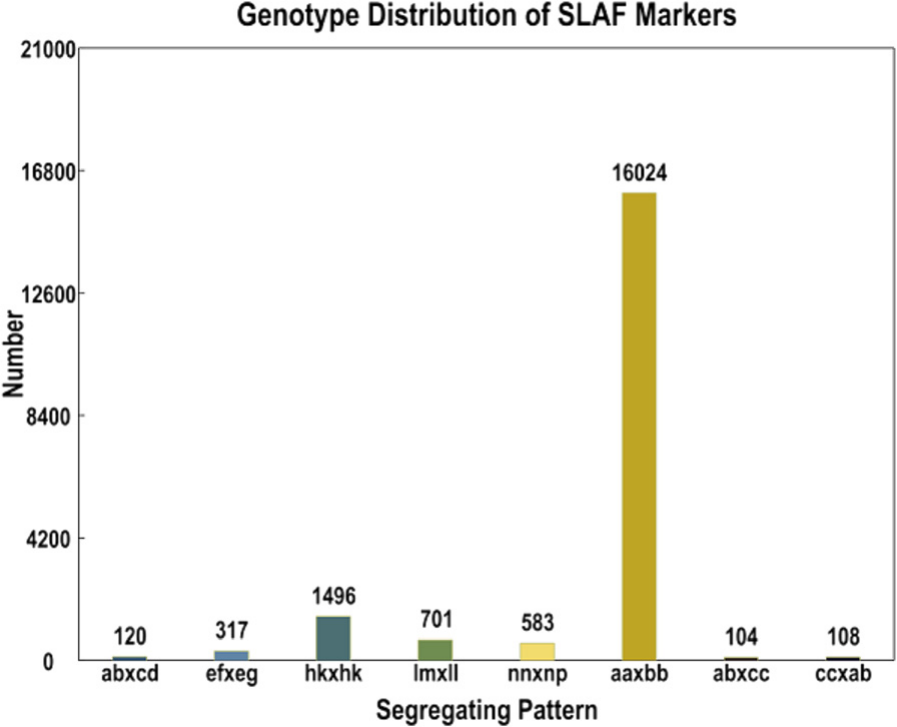
Number of SLAF markers in each of eight segregation patterns

After filtering low-depth SLAF tags, a total of 142,653 high-quality SLAFs were detected and 22,151 of them were polymorphic, with a polymorphism rate of 15.42 % (Table 1, Additional file 1). The polymorphic SLAFs were further screened to filter out unsuitable markers for genetic construction. Finally, of the 22,151 polymorphic SLAFs, 19,453 were classified into eight segregation patterns (ab × cd, ef × eg, hk × hk, lm × ll, nn × np, aa × bb, ab × cc, and cc × ab) following a genotype encoding rule (Fig. 2). Since the two parents (B214 and B227) are homozygous wax gourd lines with genotypes of aa and bb, 16,024 of the 19,453 polymorphic SLAFs with aa × bb segregation pattern were selected for linkage map construction (Fig. 2). After filtering the low quality SLAFs with parental sequence depth less than 10×, completeness less than 70 % and significant segregation distortion (p-value < 0.05), 4,607 markers were finally selected for genetic map construction.

### High-throughput linkage map construction

**Fig. 3:**
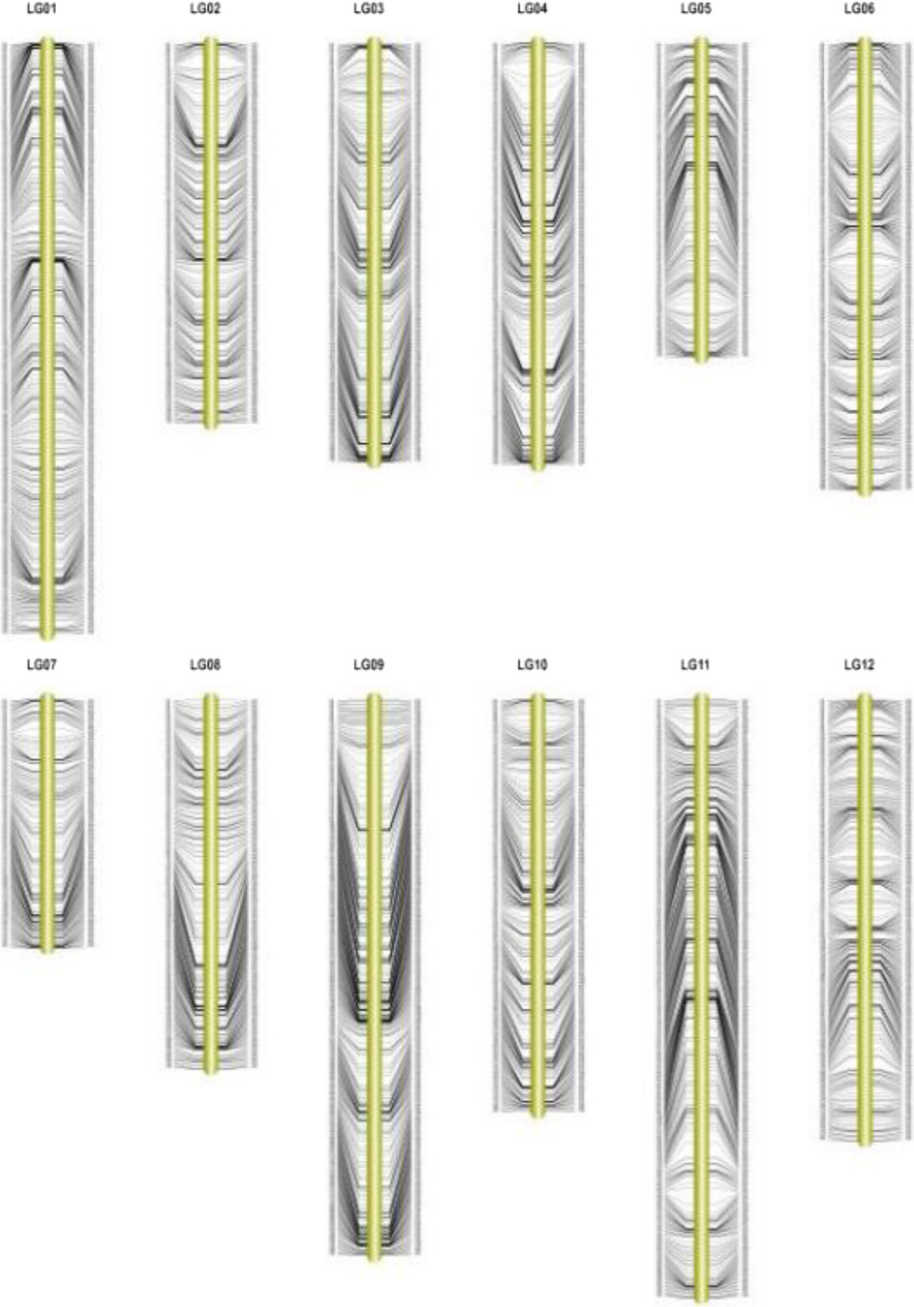
High-density genetic map of wax groud

After linkage analysis by using JoinMap 4.0 (Kyasma, NL), the 4,607 markers were mapped on twelve linkage groups (LGs) (Table 2, Fig. 3, Additional file 2). The integrity of the mapped markers ranged from 93.7 % to 98.11 %, with an average of 96.74, which indicated a relatively high map quality (Table 2). Detailed information of this genetic map is presented in Additional file 3 and Table 2.

**Table 2:** Basic information of wax gourd genetic map

Linkage	Marker number	Map length (cM)	Max distance (cM)	Marker interval (cM)	Integrity percentage (%)
LG1	524	225.20	3.68	0.43	98.05
LG2	337	151.01	5.38	0.45	96.85
LG3	371	175.27	4.37	0.47	97.76
LG4	374	151.24	6.27	0.40	97.57
LG5	278	175.16	4.80	0.63	93.70
LG6	396	160.94	3.29	0.41	96.90
LG7	220	162.42	5.56	0.74	94.68
LG8	327	196.00	4.87	0.60	93.97
LG9	493	243.32	5.16	0.49	98.11
LG10	366	149.91	3.58	0.41	97.78
LG11	530	192.81	5.64	0.36	97.94
LG12	391	189.59	4.44	0.48	97.60
Total	4,607	2,172.86	6.27	0.49	96.74

The map spanned a total of 2,172.86 cM with an average marker distance of 0.49 cM. The largest LG was LG11, which contained 530 SLAF markers. And the smallest LG7 only contained 220 markers. On average, each LG had 384 SLAF markers. The genetic distances of the 12 LGs ranged 149.91 cM (LG10) to 242.32 cM (LG9), with the average marker distance spanned from 0.36 c (LG11) to 0.74 cM (LG7) (Table 2). Besides, the max gap of the 12 LGs ranged from 3.29 cM (LG6) to 6.27 cM (LG4) (Table 2).

### Evaluation of the genetic map

To evaluate the quality of the newly constructed map, haplotype map and heat map were employed. Haplotype map reflects the double crossover of the population, which suggests the errors of genotyping. Haplotype maps, which intuitively displayed the recombination events of each individual, were generated for each of the F2 population and for the parental controls using 4,607 SLAF markers as described by West et al. [27] (Additional file 4). Most of the recombination blocks were clearly defined. In a linkage group, the percentage of double crossover is usually less than 3 %. In our present study, the percentage of double crossover of all the linkage groups is less than 0.03 % (Table 3).

**Table 3:** The double crossover and missing percent of all the linkage groups

Linkage	Double crossover percent (%)	Missing percentage (%)
LG1	0.00	1.95
LG2	0.00	3.15
LG3	0.00	2.24
LG4	0.01	2.43
LG5	0.00	6.30
LG6	0.03	3.10
LG7	0.01	5.32
LG8	0.00	6.03
LG9	0.00	1.89
LG10	0.00	2.22
LG11	0.00	2.06
LG12	0.00	2.40

Heat maps were also generated by using pair-wise recombination values for the 4,607 SLAF markers (Additional file 5). Since the heat map reflects the relationship of recombination between markers from one single linkage group, thus it was used to find potential ordering errors of markers. The result showed that the SLAF markers in most LGs were well ordered.

### Inheritance and mapping of pericarp color

**Fig. 4:**
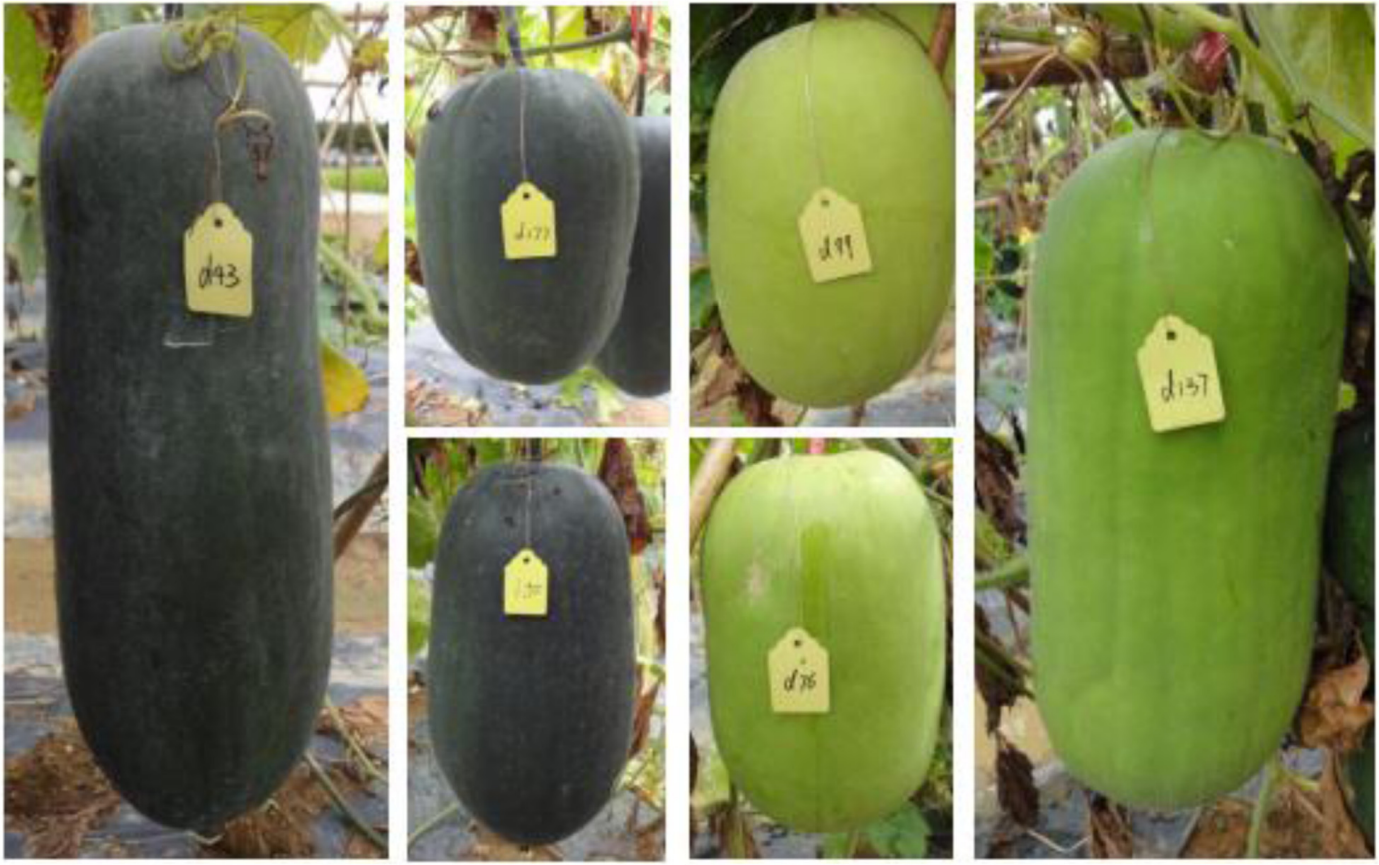
Partial fruits of F2 populations

Six generations (P1, P2, F1, F2, BC1 and BC2) were employed to study the inheritance of pericarp color. It could be easily identified on the mature fruit stage, which is yellow in P1 (B214) and dark green in P2 (B227). The pericarp color of all the F1 and BC2 plants was dark green, which indicated the dominant nature of dark green to yellow. Among the 219 F2 plants, 163 had fruits with dark green pericarp color and 56 with yellow pericarp color (*χ*2 = 0.01 for 3 dark green to 1 yellow segregation ratio) (Fig. 4). Furthermore, for 160 BC1 plants, 75 of them had dark green fruit, while the remaining had yellow fruit (*χ*2 = 0.51 for 1 dark green to 1 yellow segregation ratio). These results indicated that the pericarp color was controlled by one single gene, with dark green dominant to yellow. Herein, this gene was designated as *pc*.

**Fig. 5:**
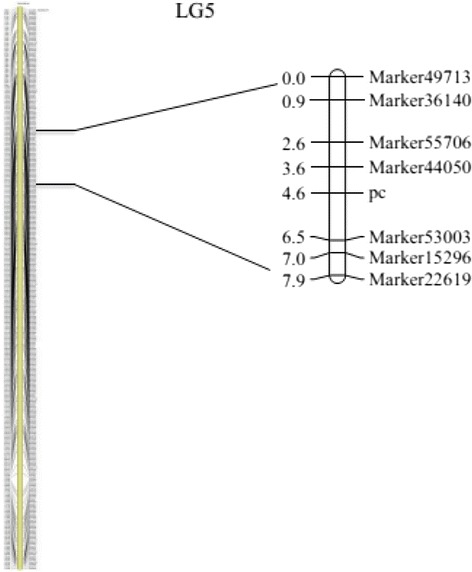
Gene mapping of pericarp color

Based on the high-density genetic map and phenotypic character, the pericarp color locus was performed by JoinMap 4.0 with the Kosambi mapping function. Threshold of significance (*P* = 0.05) for each marker after 1,000 permutation was set to 5.0, which identified a single locus at 35.6 cM on LG5 (Fig. 5). On this linkage group, 2 markers (Marker44050 and Marker53003) were found to be linked to pc locus. The genetic distance of markers in the flanking regions of *pc* was 1.0 (Marker44050) and 1.9 (Marker53003), respectively (Fig. 5). And the estimated proportion of phenotypic variance explained (PVE) by Marker44050 and Marker53003 was 82.66 % and 71.97 %, respectively.

## Discussion

High-density genetic maps play an important role in fine mapping and map-based gene cloning [16, 25, 28]. But up to recently, no genetic map has been constructed in wax gourd, although it has great economic and medicinal value. The construction of genetic map depends on efficient markers. However, the genome size of wax gourd is relatively large when compared to other species in cucurbits, and the genomic information is lack, which hinder specific markers development. Currently, only conventional markers (such as RAPD and ISSR) and a small amounts of SSR markers can be available in wax gourd [6–8]. Besides, the genetic diversity among wax gourd germplasm resources is low. The narrow genetic base results in a very low polymorphism for the existing markers, which makes it impossible to construct a high-density genetic map in wax gourd. Consequently, in order to develop high-density markers for genetic map construction, we must hunt for a more effective method.

With the rapid development of sequencing technology, NGS-based methods provide an excellent chance for high-throughput marker discovery and genotyping, which has been successfully applied in many species [29, 30]. SLAF-seq strategy is one of the newly developed methods for large-scale SNP discovery and genotyping using high-throughput sequence [20]. This method combines locus-specific amplification and high-throughput sequencing, and has been applied successfully in many plants [20–26]. As an example in sesame, Zhang et al. [21] constructed the first high-density genetic map by SLAF sequencing. In cucumber, two saturated genetic map were generated by using SLAF-seq technology, and QTL analysis of fruit-related traits were also conducted [25, 26].

When compared with other methods for marker development, the SLAF-seq strategy had several significant advantages, such as uniform, accuracy, stability, high success rate, and low cost. Furthermore, reference genome sequences or reference SNPs were not necessary for this method [20]. Consequently, SLAF-seq is an ideal strategy for large-scale marker developing and genotyping for species which is lack of reference genome information, such as wax gourd. In the present study, we firstly employed SLAF sequencing technology in wax gourd. In total, 143.38 M pair-end reads and 142,653 high-quality SLAFs were generated. After filtering low-depth SLAF tags, 22,151 of high-quality SLAFs were polymorphic, with a polymorphism rate of 15.42 %. Eventually, 4,607 polymorphic markers were identified for constructing genetic map, which contained 12 LGs, with the number of markers ranged from 220 to 530 (Table 2, Fig. 3). The map spanned 2,172.86 cM with an average marker distance of 0.49 cM. In order to evaluate the quality the genetic map, haplotype map and heat map were applied. The result showed that the SLAF markers in most LGs were well ordered, which implied that the quality of markers fulfilled the requirements for genetic map construction. Besides, marker integrity and accuracy were high. The above results also accurately reflect the genetic and polymorphism characteristics of wax gourd.

Pericarp color is an important trait for fruit commodity value, which has been studied in many species. Cheng et al. [31] investigated the inheritance of apple fruit skin color and identified DNA markers linked to the gene for this trait. And two allelic *R2R3 MYB* genes *MdMYBA* and *MdMYB1* were isolated from a pale-red skinned apple cultivar and a red skinned apple cultivar, respectively [32, 33]. In Japanese pear, a major QTL associated with fruit skin color was identified at the top of LG8 [34]. The skin color in sweet cherry fruit was also reported, which identified a QTL on LG6 [35]. Huh et al. [36] identified a candidate gene, which may be responsible for discriminating red and orange pepper. In *Cucurbitaceae*, the mature fruit color of cucumber is controlled by one single gene, which has been fine mapped on chromosome 4 [37].

In this study, we researched the genetic inheritance of pericarp color by using 6 generations of wax gourd, which showed that the pericarp color was controlled by one single gene, with dark green dominant to yellow. And we also conducted gene mapping analysis using the high-density genetic map. We identified a single locus on LG5. And the genetic distance of the flanking markers of *pc* locus was 1.0 (Marker44050) and 1.9 (Marker53003), respectively (Fig. 5). Furthermore, the estimated PVE contribution rate by these two markers was 82.66 % and 71.97 %, respectively. These results indicate that the high-density genetic map developed in this study can be successfully employed for gene mapping.

## Conclusion

In the present study, we generated 143.38 M pair-end reads and 142,653 high-quality SLAFs using SLAF sequencing. A high quality genetic map was constructed based on 4,607 markers. The genetic length of this map was 2,172.86 cM, with an average marker distance of 0.49 cM. Haplotype map and heat map were employed to evaluate the quality of this genetic map. Based on haplotype map, the percentage of double crossover of all the linkage groups was under 0.03 %. And the heat map showed the SLAF markers in most LGs were in well order. In addition, we analyzed the inheritance of pericarp color, which showed that the pericarp color was controlled by one single gene. We further applied the newly constructed map to identify a single locus on chromosome 5 for controlling the pericarp color. To the best of our knowledge, both the genetic map construction and gene mapping of pericarp color are the first reports in wax gourd. The high-density map constructed herein will be served as a valuable tool for gene mapping, marker assisted breeding, map-based gene cloning, comparative mapping and draft genome assembling in wax gourd.

## Methods

### Plant materials and DNA extraction

Two wax gourd inbred materials, ‘B214’ and ‘B227’, were employed in our present study. ‘B214’ has small mature fruit with yellow skin color, while ‘B227’ has big mature fruit with dark green skin color. In order to investigate the inheritance of pericarp color, ‘B214’ was crossed with ‘B227’, and then the F1, F2, BC1 and BC2 were generated. And the F2 population which consisted of 140 individuals was used for genetic map construction.

Seedlings of the offspring and parents were planted in the research experiment field of Vegetable Research Institute, Guangdong Academy of Agricultural Sciences, Guangzhou, China. Young healthy leaves from the two diploid parents as well as F2 individuals were frozen in liquid nitrogen immediately, and stored at −80 °C freezer. Total genomic DNA was extracted from each leaf sample using the Cetyltrimethyl ammonium bromide (CTAB) method [38]. DNA quality was evaluated by electrophoresis in 1 % agarose gel, and the concentration was estimated with a NanoDrop 2000C Spectrophotometer (Thermo scientific, USA).

### SLAF library preparation and high-throughput sequencing

The procedure of SLAF library construction was performed as described by Sun et al. [20] with minor modifications. A pilot experiment was designed to evaluate the enzymes and sizes of restriction fragments to produce large number and high-quality SLAFs. Four criteria were considered. i) The proportion of SLAFs locating in the repeat regions must be as low as possible. ii) The SLAFs must be evenly distributed in the genome. iii) The length of SLAFs must be suitable for specific experimental system. iv) The final number of SLAFs must meet the expected one. Based on the result of the pilot experiment, the SALF library was constructed as following procedures. Firstly, Genomic DNA was digested by Hpy166II (NEB). Subsequently, a single nucleotide (A) overhang was added to the digested fragments. Then dual-index sequencing adapters were ligated to the A-tailed fragments using T4 ligase (NEB). Polymerase chain reaction (PCR) was carried out in the reaction solutions containing the diluted restriction-ligation DNA samples, dNTP, Taq DNA polymerase (NEB) and PCR primers containing barcode 1. The products were then purified and separated on a 2 % agarose gel. Fragments ranging from 264 to 314 bp (with indexes and adaptors) in size were excised and purified using a Gel Extraction Kit (Qiagen, Hilden, Germany). Subsequently, these fragments were subjected to PCR amplification with to add barcode 2. The samples were then gel purified, and diluted for sequencing. Pair-end sequencing was performed on an Illumina HiSeq 2500 system (Illumina, Inc., San Diego, CA, USA) according to the manufacturer’s recommendations.

### SLAF-seq data grouping and genotyping

The SLAF-seq data grouping and genotyping were performed following the procedures described by Sun et al. [20]. All SLAF pair-end reads with clear index information were clustered according to sequence similarity, which was detected by BLAT (−tileSize = 10 –stepSize = 5) [39]. Sequences with over 95 % identity were considered as identity ones and grouped in one SLAF locus as described by Sun et al. [20]. Single nucleotide polymorphism (SNP) loci of each SLAF locus were then detected between parents, and SLAFs with more than 3 SNPs were filtered out firstly. Minor allele frequency (MAF) evaluation was performed to define alleles in each SLAF. Since wax gourd is a diploid species, one locus can contain up to four SLAF tags, consequently, groups containing more than four tags were considered as repetitive SLAFs and filtered out. In our present study, SLAFs with a sequence depth of less than 10 were defined as low-depth SLAFs and filtered out in the following analysis. Polymorphic SLAFs containing 2 ~ 4 tags were considered as potential markers. Polymorphic SLAF markers were classified into eight segregation patterns as following: ab × cd, ef × eg, hk × hk, lm × ll, nn × np, aa × bb, ab × cc, and cc × ab). Since the F2 population was derived from two fully homozygous parents with a genotype of aa or bb, so only the SLAF markers with segregation patterns of aa × bb were used for genetic map construction.

### Linkage map construction and mapping of pericarp color

The modified logarithm of odds (MLOD) scores was calculated for the high-quality SLAFs from the F2 population, which can be used for linkage clustering. Prior to ordering, markers with MLOD scores <5 were filtered. Subsequently, HighMap software [40] was employed to genetic map for each linkage. The software constructs high-quality genetic map base on efficient maximum likelihood estimation method. Kosambi mapping function was employed to convert recombination percentages to genetic distance in cM. Furthermore, haplotype map and heat map were used to evaluate the quality of the constructed linkage map.

The phenotypic data on pericarp color was collected. Chi-square tests for goodness-of-fit were used to test for deviations from the expected 3:1 segregation ratio in F2 population and 1:1 segregation ratio in BC1 population. Linkage analysis of the pericarp color loci with SLAF-markers was performed with the Kosambi mapping function using JoinMap 4.0 (Kyasma, NL).

## Additional files

Additional file 1:
**Total SLAFs.** (XLSX 16699 kb) Additional file 2:
**Sequence information of the mapped SNP markers.** (XLSX 657 kb) Additional file 3:
**Detailed SNP marker information for each of the twelve linkage groups.** (XLSX 113 kb) Additional file 4:
**Haplotype maps of the genetic map.** (PDF 1005 kb) Additional file 5:
**Heat maps of the genetic map.** (PDF 363 kb)

## Abbreviations

CTAB: Cetyltrimethyl ammonium bromide
ISSR: Inter-simple sequence repeat
LG: Linkage group
MAS: Marker-assistant selection
MAF: Minor allele frequency
MLOD: Modified logarithm of odd
NGS: Next-generation sequencing
pc: pericarp color
PCR: Polymerase chain reaction
PVE: Phenotypic variance explained
RAPD: Random amplified polymorphic DNA
RRGS: Reduced representation genome sequencing
SLAF: Specific-locus amplified fragments
SNP: Single-nucleotide polymorphism
SSR: Simple sequence repeats
