# Supplementary figures and images for: High-density genetic map construction and gene mapping of pericarp color in wax gourd using specific-locus amplified fragment (SLAF) sequencing
Source: BMC Genomics. 2015 Dec 9;16:1035. doi: 10.1186/s12864-015-2220-y (PMC4673774; doi:10.1186/s12864-015-2220-y)

# Haplotype maps of the genetic map.

Chr1

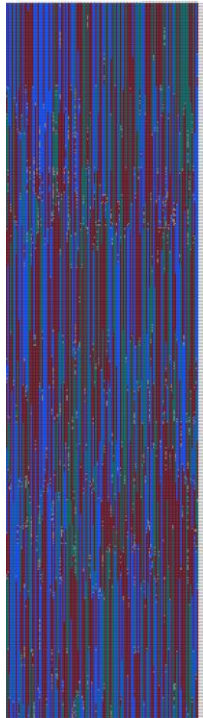

Chr2

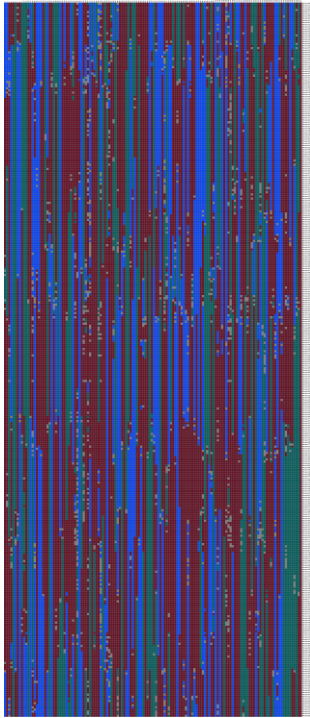

Chr3

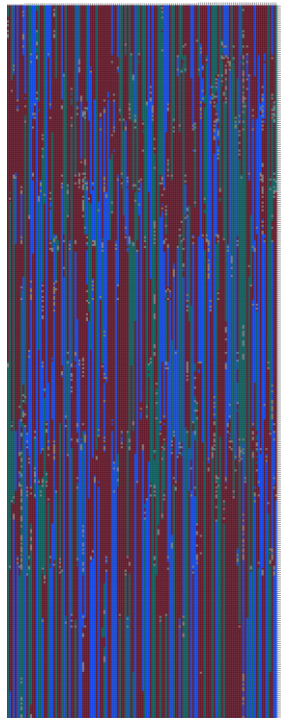

Chr4

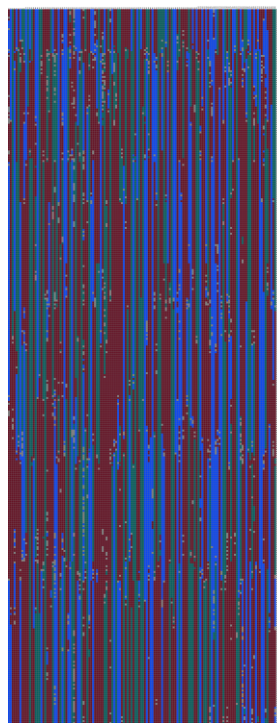

Chr5

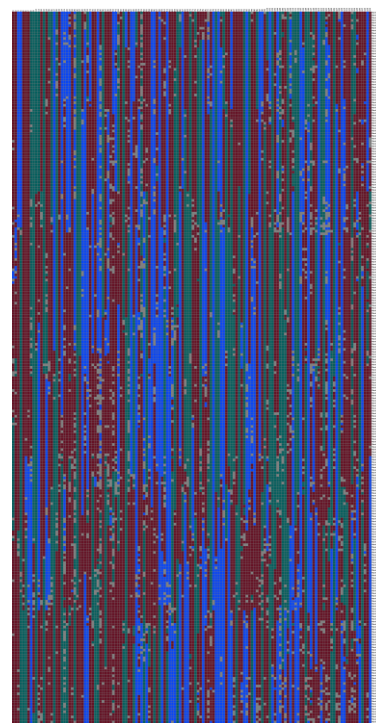

Chr6

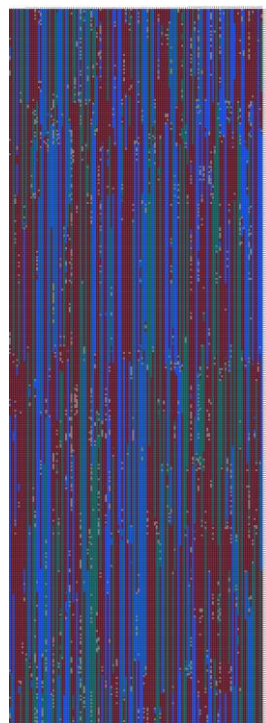

Chr7

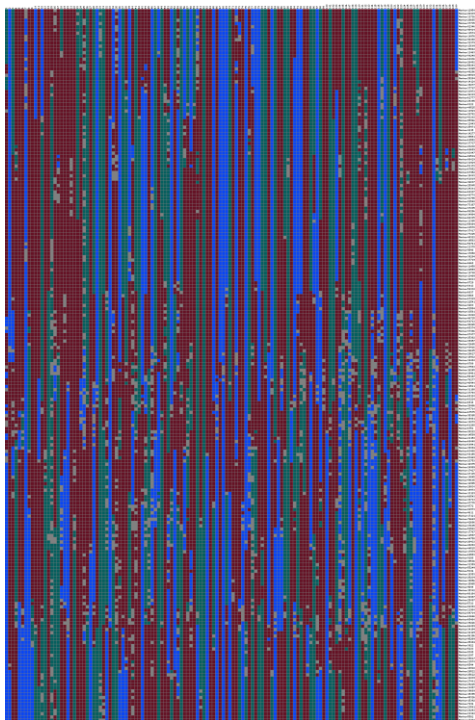

Chr8

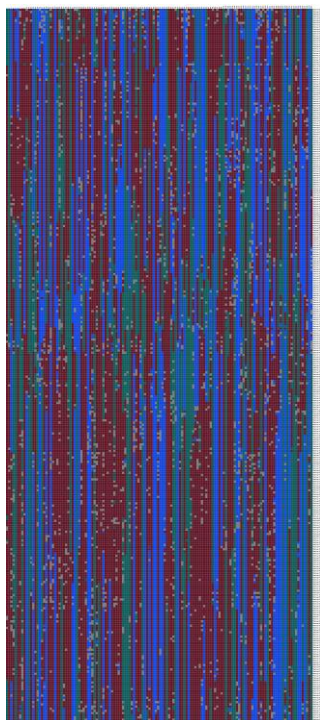

Chr9

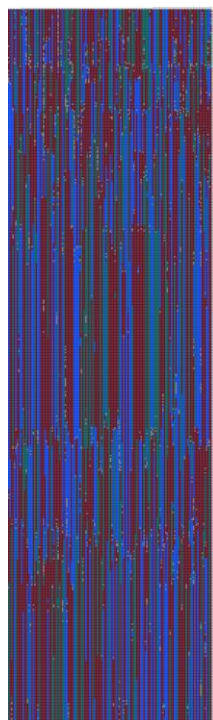

Chr10

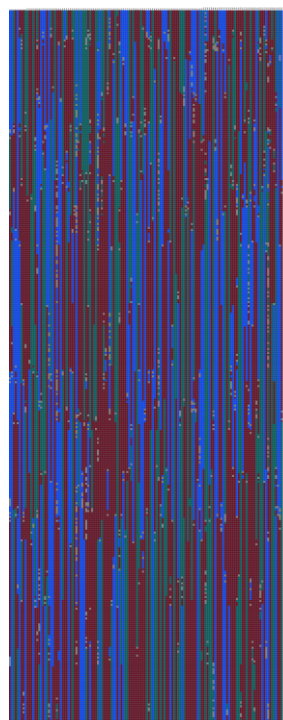

Chr11

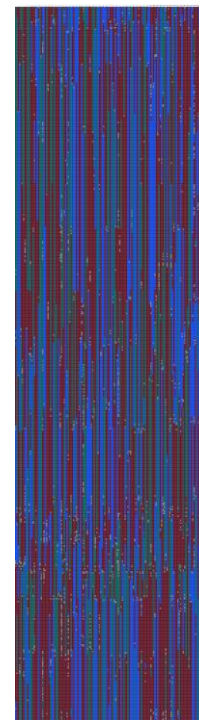

Chr12

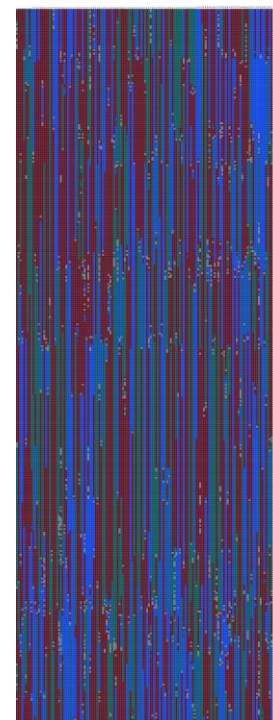

Supplement: Additional file 4: — Haplotype maps of the genetic map. (PDF 1005 kb) [file 12864_2015_2220_MOESM4_ESM.pdf]

## Heat maps of the genetic map

Chr 1

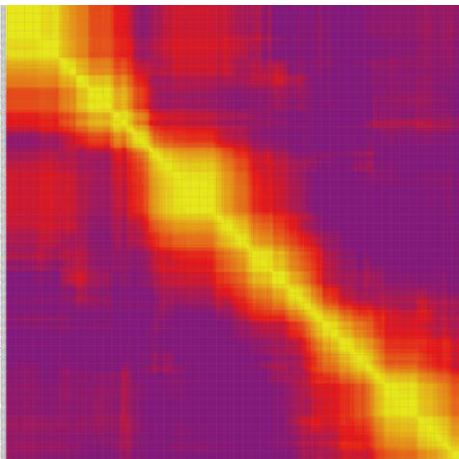

Chr 2

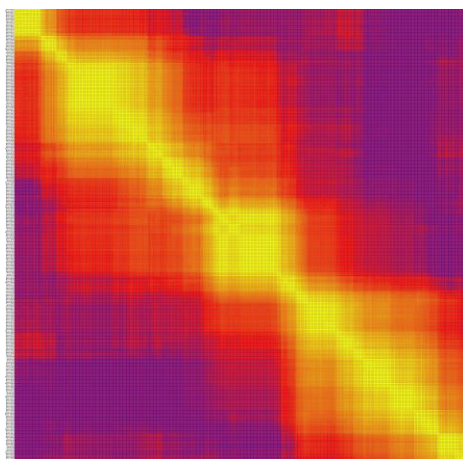

Chr 3

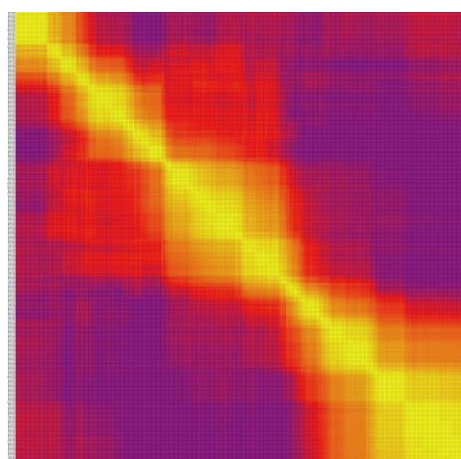

Chr 4

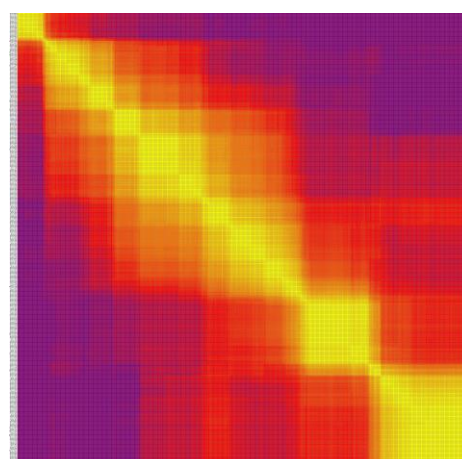

Chr 5

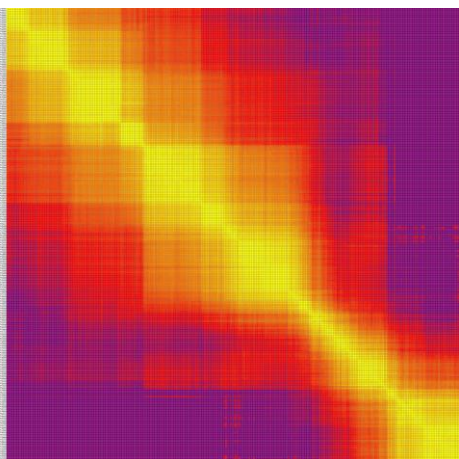

Chr 6

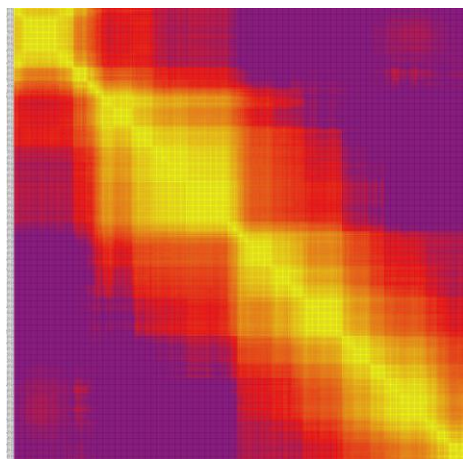

Chr 7

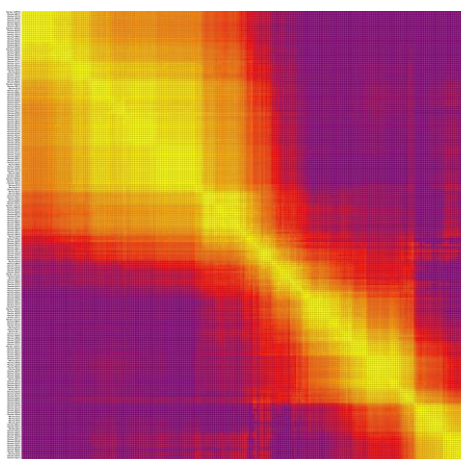

Chr 8

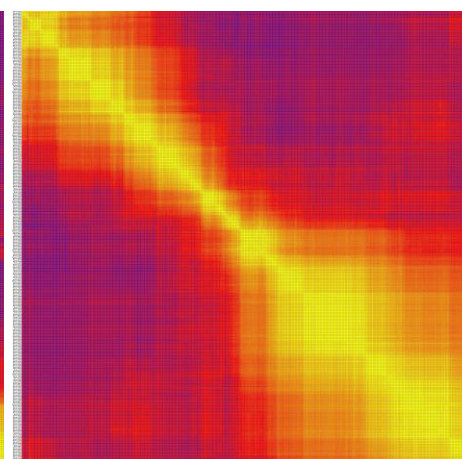

Chr 9

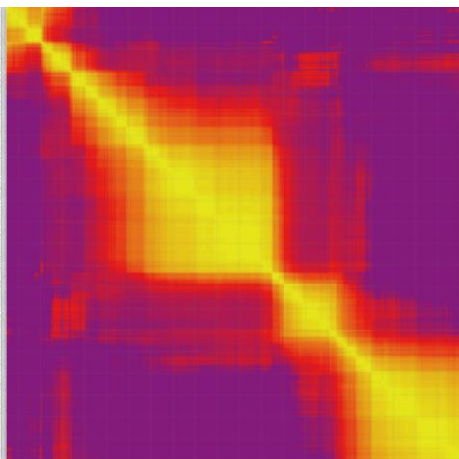

Chr 10

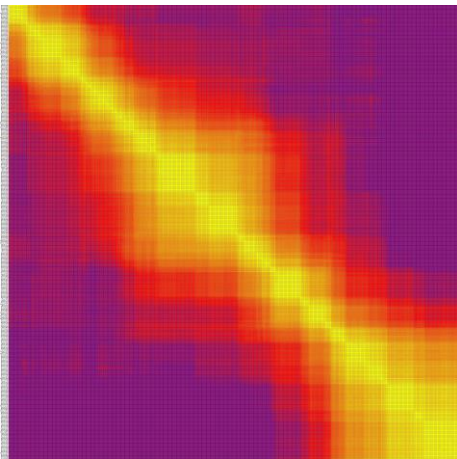

Chr 11

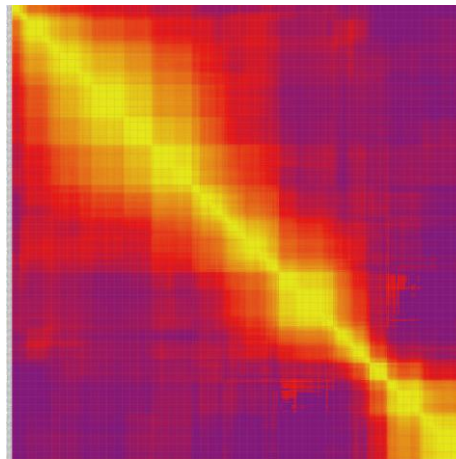

Chr 12

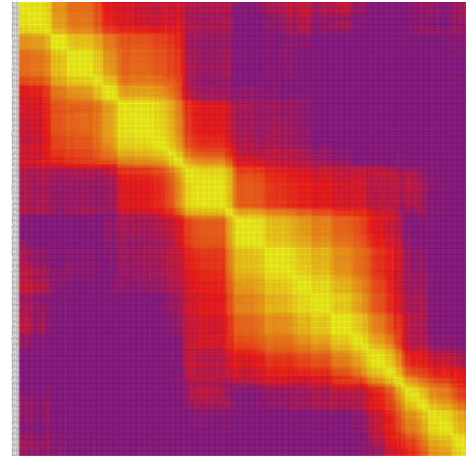

Supplement: Additional file 5: — Heat maps of the genetic map. (PDF 363 kb) [file 12864_2015_2220_MOESM5_ESM.pdf]
